# There is more to life than serum vitamin D: a lesson from the past

**DOI:** 10.1042/CS20211176

**Published:** 2022-04-27

**Authors:** Martin Hewison

**Affiliations:** Institute of Metabolism and Systems Research, University of Birmingham, Birmingham B15 2TT, U.K.

**Keywords:** 25-hydroxyvitamin D, adipose, bile, cholecalciferol, metabolism

## Abstract

This commentary revisits a paper from Clinical Science in 1972 entitled “The distribution and storage of vitamin D and its metabolites in human tissues” by Barbara Mawer, Bill Stanbury and colleagues. The paper continues to be well cited 50 years later, in part because the study it describes – which includes the use of human autopsy tissue – would be difficult to replicate today. However, the paper also has resonance today because the focus of the study – what is the fate of vitamin D in the body? – is still not clear. This commentary discusses why the Mawer et al. study was a major advance when published and why there is still much to be learned from this paper half a century later.

This year is the 100th anniversary of the first use of the term vitamin D to describe a fat-soluble factor that cured rickets in dogs [1,2]. The 50 years that followed this saw rapid advances in the chemistry of vitamin D that included discovery of the synthesis of vitamin D by the action of ultraviolet light on skin [3], and the subsequent isolation of vitamin D (cholecalciferol) [4] and 25-hydroxvitamin D (25(OH)D) [5], the major circulating form of vitamin D. The last 50 years have witnessed equally remarkable changes in vitamin D research. During the 1970s and 1980s landmark discoveries such as isolation of the active form of vitamin D, 1,25-dihydroxyvitamin D (1,25(OH)_2_D) [6,7], shifted the focus on vitamin D from nutrition to endocrinology and paved the way for vitamin D research that paralleled work on other steroid hormones. The last 20 years of vitamin D research have been characterised by yet another change in the direction of vitamin D research, switching the emphasis back to vitamin D nutrition, with myriad association studies linking serum 25(OH)D levels with diverse human diseases, and the advent of large-scale trials to assess the possible health benefits of vitamin D supplementation beyond its established actions in protecting against rickets. In 100 years, the vitamin D initially described by Elmer McCollum and Edward and May Mellanby has changed beyond all recognition – the recent discussion in the UK Parliament of the possible benefits of vitamin D supplementation for COVID-19 illustrates this nicely. What will the next 100 years of vitamin D research look like, and how will this compare to the first 100 years? Like many other areas of biomedical science there is still much to be learned from the history of vitamin D research. This commentary looks back at a vitamin D paper that was published in Clinical Science 50 years ago but continues to inform researchers today and may yet provide some important pointers for vitamin D research in the future.

Despite the many different facets of modern-day vitamin D, our view of the impact of vitamin D on human health continues to be influenced by seminal studies that provide a mechanistic rationale for the clinical applications of vitamin D. In 1972, precisely midway through the first “Vitamin D Century”, Barbara Mawer, Bill Stanbury and colleagues published a study in Clinical Science on “The distribution and storage of vitamin D and its metabolites in human tissues” [8] that perfectly illustrates the importance of fundamental vitamin D biology in providing a foundation for future research. In this case, Mawer et al. quantified the uptake of vitamin D by human tissues, showing that sites such as adipose tissue and muscle can be reservoirs for vitamin D metabolites outside the circulation. The study also described new concepts in the analysis and biochemistry of vitamin D that foreshadowed the rapid development of liquid chromatography and immunoassays and the associated dramatic expansion of vitamin D research in the late 1970s. Fittingly this also coincided with the stellar careers of Barbara Mawer and Bill Stanbury who, along with competitors such as my own mentor Jeffrey O’Riordan, helped to maintain the UK at the centre of the vitamin D research world. The manuscript is a fascinating read on multiple levels. The most obvious thing that stands out from this study is that ^3^H or ^14^C-labelled cholecalciferol was administered to human subjects, and subsequent tissue distribution analysed, an approach previously pioneered by the same group in another Clinical Science publication [9]. The difference with the 1972 paper was that subsequent analysis of the distribution of ^3^H or ^14^C-labelled cholecalciferol was carried out using tissue either surgically removed or, in the majority of cases, obtained following autopsy. The authors indicate that the participants, who were all seriously ill, gave their consent to these procedures but, nevertheless, it seems unlikely that current ethical scrutiny would sanction similar studies today. Indeed the nature of this study as a whole contrasts starkly the rigorous Safety and Monitoring oversight of even a low level vitamin D supplementation trial in the modern era.

For the analytical chemist the Mawer et al. paper provides the first description of the use of tissue extraction protocols initially described by Lund and DeLuca [10] and Bell and Kodicek [11] in combination with Barbara Mawer’s own methodology for silicic acid column separation of vitamin D metabolites [12]. The development of high-performance liquid chromatography (HPLC) technology that followed on from this was an important factor in the key advances that defined vitamin D research at the end of the 20th Century. Mawer et al. utilised new tissue extraction and chromatography techniques to assess the tissue distribution of vitamin D_3_ (cholecalciferol) and 25(OH)D_3_ fractions following administration of radiolabelled cholecalciferol. However, tucked away at the end of the Materials and Methods section, is a description of a “Biological assay” used in the study. Although brief, this is one of the more fascinating parts of the Mawer et al. paper in that it represents one of the last connections with the early days of vitamin D research. Specifically, the assay used was an *in vivo* biological assay that assessed the capacity for extracts of human serum to cure rickets in young Wistar rats. This methodology is described in greater detail in another manuscript by Barbara Mawer [13], but in the Clinical Sciences paper there is relatively little information other than a sentence that states – “*Because of the frequent need to make replicate assays on individual tissues, approx. 1300 rats were used to produce the data of Table 4*”. How would this fare under 3Rs scrutiny in the 21st century? The *in vivo* strategy utilised by Mawer et al. was, of course, a throwback to the early studies of McCollum [2] and Mellanby [1] and preceded by a few years the development of competitive protein binding assays [14] and radioimmunoassay technology [15] that would revolutionise not only vitamin D research but also the whole field of endocrinology.

Despite the vast numbers of rats required for this part of the study, the authors were able to show that the distribution of vitamin D metabolites determined by analysis of tissue radioactivity following administration of radiolabelled cholecalciferol could also be observed when assessing biological vitamin D activity in these tissues. The study highlights adipose tissue and muscle as potential reservoirs of vitamin D, notably in the setting of excess vitamin D, confirming previous proposals for tissue storage of vitamin D that were based on the observation of limited excretion of vitamin D following supplementation dosing. The fact that Mawer et al. continues to be cited today reflects the continued interest in the tissue fate of vitamin D – notably in the setting of the many supplementation trials that have characterised 21st century vitamin D research. This is due, in part, to our current understanding of how vitamin D functions. The hormonal view of vitamin D that was established a few years after the Mawer et al. Clinical Science paper has now been expanded to include autocrine/paracrine models that incorporate tissue acquisition of 25(OH)D, endogenous synthesis of 1,25(OH)_2_D via the enzyme 25-hydroxyvitamin D-1α-hydroxylase (1α-hydroxylase) and interaction with the nuclear vitamin D receptor (VDR) in the same cell [16].

Tissue-specific handling of vitamin D is now at the very heart of the many association studies that have linked low vitamin D (serum 25(OH)D) status with impaired health outcomes for common cancers, autoimmune and infectious diseases and neurological disorders [17]. Moreover, every vitamin D supplementation study carried out in the last 20 years has automatically assumed that serum elevation of 25(OH)D is paralleled by tissue-specific uptake of 25(OH)D and autocrine conversion to 1,25(OH)_2_D. The Mawer et al. paper endorses this general concept, but it also sends out a strong warning to anyone making this broad assumption. The levels of vitamin D bioactivity in different tissues and different individuals varied significantly, which may be due to the fact the participants in this study were very ill and died shortly after administration of radiolabelled vitamin D. Despite this limitation, the resulting access to autopsy material meant that the authors were able to demonstrate that adipose tissue and muscle are major pools of vitamin D in the human body. With the increasing prevalence of obesity in communities around the world, it is unclear whether a particular dose of vitamin D supplementation will achieve the same tissue distribution of 25(OH)D in people with high versus low fat mass. Many recent publications have described lower serum levels of 25(OH)D in subjects with higher fat mass [18], supporting the proposal that adipose tissue can act as a reservoir for lipophilic 25(OH)D. The study by Mawer et al. suggests that tissues such as fat and muscle can retain significant amounts of 25(OH)D for many days following administration of supplementary cholecalciferol. The precise mechanisms that promote the release of 25(OH)D and other vitamin D metabolites from these tissues are still unclear, but it is interesting to note a recent report indicating that physical activity may play a role in this process. In the study by Dzik et al., a single bout of exercise in young subjects was sufficient to elevate serum levels of 25(OH)D [19].

The second facet of the Mawer et al. paper that garnered less attention in subsequent years is the investigation of vitamin D excretion in bile. The paper reported that some cholecalciferol and 25(OH)D was excreted in bile but the predominant excreted forms of vitamin D were ‘polar metabolites’. We now know that these are most likely conjugated forms of vitamin D – 25(OH)D-3-O-sulfate (25(OH)D-S) and 25OHD-3-O-glucuronide (25(OH)D-G). Remarkably, relatively little is known about the biological function of conjugated forms of 25(OH)D, although recent studies have shown that 25(OH)D-S is present in abundance in serum, circulating at levels similar to 25(OH)D itself [20]. Fifty years after the Mawer et al. paper, we still know little about conjugated forms of vitamin D and their potential biological activity beyond their presence in bile. Given the importance of conjugated metabolites for other steroid hormone systems [21], this should be a priority for future vitamin D research, particularly in the context of the many expensive and time-consuming supplementation trials that have relied on oral doses of cholecalciferol without any clear knowledge of where the metabolites of cholecalciferol will end up. Indeed, one could make a strong case for a re-run of the Mawer and Stanbury study, albeit in the setting of a more acceptable protocol!

Revisiting this study from Clinical Science 50 years ago was carried out against the backdrop of all the developments that have occurred in the subsequent 50 years of vitamin D research. As outlined earlier in this review, the paper by Mawer et al. is a perfect representation of research at the very frontier of the old and new worlds of vitamin D – it paid homage to discoveries in the 1920s whilst anticipating the new research that was still to come. Can a study such as this continue to be relevant as we move into the second century of vitamin D? Mawer et al. has been cited almost 400 times (including major guidelines and reviews [22,23]) but 85 of these were in the last 5 years. Interest in vitamin D is at an all-time high, and the technologies available to vitamin D researchers are far beyond what might have been anticipated even in 1972. The availability of genetically manipulated animal models, coupled with single cell transcriptomic/metabolomics and *in vivo* imaging technologies have greatly increased our capacity to define the fate of biological molecules such as vitamin D. In the field of vitamin D metabolism it is now clear that there are multiple forms of vitamin D beyond those envisaged by Mawer et al., with liquid chromatography tandem mass spectrometry methods that can simultaneously measure all of these metabolites in a single serum sample [24]. Most recently, new Mass Spectrometry Imaging technology has been described that enables physical mapping and quantification of steroid hormone-like molecules in complex tissues. Ground-breaking work by Diego Cobice and colleagues has shown how this can be used to measure the precise distribution of vitamin D metabolites in tissue sections [25]. These new strategies for analysis of vitamin D will have a major impact on the future of vitamin D research but it will be interesting to see if these developments have the lasting impact on research that is so clearly demonstrated in the work of Mawer et al. (1972) in Clinical Science.

## Abbreviations

HPLC: high-performance liquid chromatography
VDR: vitamin D receptor
